# Changes in Reproductive Traits in *Physalis philadelphica*; An Unexpected Shift Toward Self-Incompatibility in a Domesticated Annual Fruit Crop

**DOI:** 10.3389/fpls.2021.658406

**Published:** 2021-05-21

**Authors:** Lislie Solís-Montero, Lorena Aceves-Chong, Mayumi Vega-Polanco, Ofelia Vargas-Ponce

**Affiliations:** ^1^Laboratory of Pollinator, Pest and Vector Arthropods, Department of Agriculture, Society and Environment, El Colegio de la Frontera Sur, Tapachula, Mexico; ^2^Consejo Nacional de Ciencia y Tecnología, Mexico City, Mexico; ^3^Centro de Investigación y de Estudios Avanzados del Instituto Politécnico Nacional, Irapuato, Mexico; ^4^Departamento de Botánica y Zoología, Instituto de Botánica, Centro Universitario de Ciencias Biológicas y Agropecuarias, Universidad de Guadalajara, Zapopan, Mexico

**Keywords:** autonomous autogamy, domestication, commercial-plants, husk tomato, landraces, plant reproduction, *Physalis philadelphica*, weedy populations

## Abstract

Domestication is an evolutionary process with an impact on plant reproduction. Many domesticated plants are self-compatible (i.e., they lack mechanisms to reject their own pollen), but few domesticated plants are fully or partially self-incompatible. We used the husk tomato, *Physalis philadelphica*, as a study model to investigate changes in the reproductive strategy of an annual partially self-incompatible plant during the process of domestication. Wild and cultivated populations of this species coexist in close proximity. These different populations present a high level of morphological and genetic variation associated with different degrees of domestication. We hypothesized that artificial selection favors self-compatibility in cultivated plants through changes in their reproductive strategy and some reproductive parameters associated with domestication. To test this hypothesis, we characterized the floral morphology and some reproductive parameters of weedy plants (wild plants), landraces (semi-domesticated plant), and commercial plants (domesticated plants). We conducted an artificial crossing experiment, germinated the seeds, and recorded seedling growth. Commercial plants had the largest flowers and the highest number of ovules. Yet, they did not differ in other reproductive parameters (e.g., herkogamy, size of pollen grains, stigmatic area, and pollen:ovule ratio) from landraces and weedy plants. *Physalis philadelphica* produced fruits by autonomous autogamy in the artificial crossing experiment. These fruits were the smallest and lightest fruits at all degrees of domestication; however, fruit set of autonomous autogamy was higher in weedy plants. In addition, fruit production was higher when weedy plants donated pollen to commercial plants. Although seeds produced by autonomous autogamy of weedy plants had a low germination percentage, their cotyledons and the embryonic foliage leaves appeared earlier than in landraces and commercial plants. In conclusion, the domestication syndrome in this plant was manifested as increments in flower size and ovule production. Contrary to expectations, there was higher fruit production by autonomous autogamy in weedy plants than in cultivated plants. It seems that artificial selection in *P. philadelphica* favors self-incompatibility in cultivated plants. Nonetheless, spontaneous self-pollination seems to be advantageous in weedy populations because they produced viable seeds from which cotyledons and the embryonic foliage leaves emerged earlier than in cultivated plants.

## Introduction

Plant domestication is an evolutionary process mediated by artificial human selection and evolutionary forces, which continues to act on managed or cultivated plant populations (Casas and Barbera, 2002). This process can modify not only reproduction and survival of domesticated plants, but also genetic composition and ecological behavior (Bye and Linares, 2000; Pickersgill, 2007). That is why it is possible to find different degrees of plant domestication, which vary phenotypically and genotypically in the same species (Purugganan and Fuller, 2009).

Populations of a plant species can present a gradient of modifications. (1) Domesticated populations are crops that vary in terms of morphology and genetics from wild relatives. They depend on humans to survive as they have lost their ecological adaptations. (2) Despite being cultivated, semi-domesticated populations are able to survive in the wild without human intervention, but their selective traits are different from those observed in wild populations. (3) Incipiently domesticated populations, for example, are cultivars whose selected traits do not differ from those found in wild populations. (4) Adapted to human-disturbed environments, incidentally co-evolved populations display no human selection trait. (5) And, wild- or non-domesticated populations are natural populations, whose genotype and phenotype have not been modified by humans (Clement, 1999; Meyer et al., 2012). When a plant species becomes the targeted of artificial selection, crops are classified in different evolutionary stages regarding their diversification and phenotypic characteristics (Meyer and Purugganan, 2013). In the Pre-stage 1, deliberate planting or attendance of wild plants that have favorable traits occurs. In the Stage 1, after attempts to develop or establish cultivation, crop selection occurs in an agroecosystem, which leads to the onset domestication. In Stage 2 there is an *in situ* increase of frequencies of desirable alleles in the population that leads to initial increase in yield, and selection favors crop phenotypes increasing trait variation. In Stage 3, the domesticated crop extends its geographic ranges and adapts to new environments. Finally, in Stage 4 deliberate breeding of crop varieties aims to maximize yield, ease farming effort, and increases uniformity and quality.

During domestication, artificial selection decreases fitness in natural conditions, but increases fitness to human exploitation (Meyer and Purugganan, 2013). Therefore, traits selection affects reproductive success or plant defenses (Rosenthal and Dirzo, 1997; Meyer et al., 2012). Early plant breeders have observed some characteristics exhibited by plants or populations that facilitate domestication, such as self-pollination, vegetative propagation, and delayed seed dispersal (Cox, 2009). For this reason, most crops (75%) are self-compatible (SC) (lack of mechanisms to reject their own pollen). Only a few crops have self-incompatibility (SI) systems, and a few others have partial or complete loss of the SI system (Dempewolf et al., 2012). Another group of crops have changes in their reproductive strategy due to their level of polyploidy (Meyer et al., 2012), which leads to partial sterility and promotes agamospermy (Richards, 1997). In the case of SI species, artificial selection favors SC in order to select rapidly and achieve cultivar uniformity of a desirable phenotype is associated with the costs of inbreeding (Rick, 1988; Bixby and Levin, 1996). This change in the mating system is sometimes accompanied by changes in floral morphology that favor self-pollination such as reduction in floral size and in stigma exertion (Rick, 1988).

The Solanaceae is one of the main plant families with many domesticated species. Its large family comprises 2,700–3000 species widely distributed in tropical and subtropical regions around the world, with most of the species concentrated in America (Nee, 1993; Olmstead et al., 2008; Särkinen et al., 2013). Many solanaceous species belonging to the genera *Capsicum* and *Solanum* have been subjected to artificial selection for food production on a global or regional scale. For example, other Solanaceae genus, as *Physalis*, possesses attractive characteristics in its edible fruit to local consumption. Also, it has been reported that the Solanaceae display an RNase-based SI gametophytic system (i.e., RNases as the mechanism of self-pollen recognition and rejection), which was the ancestral state in most eudicots (Igic and Kohn, 2001). In the Solanaceae SI has been lost a minimum of 60 times (Igic et al., 2006). Once the SI system is lost, it is irreversible (Igic et al., 2003, 2006). For example, in *Solanum* sect. *Lycopersicon* a recent transition to SC has been documented (Igic et al., 2008). The cultivated tomato, *Solanum lycopersicum*, is considered SC but its wild relatives are SI, or have a mixed mating system (Igic et al., 2008; Bedinger et al., 2010).

Nowadays, it is estimated that 39% of Solanaceae species are SI (Igic et al., 2006), the rest of the species are SC (e.g., *Capsicum*: Pickersgill, 1997) or exhibit a transition to SC with diverse mating systems (e.g., *Solanum* sect. *Lycopersicon*; Bedinger et al., 2010). The Solanaceae, thus, provides an excellent opportunity to study the transition from SI to SC as related to artificial selection. In particular, one interesting study model is *Physalis philadelphica* Lam. (Solanaceae). This husk tomato is a partially self-incompatible plant that was domesticated in Mexico, where it is important to both economy and culture (e.g., in Mexican gastronomy; Montes-Hernández and Aguirre-Rivera, 1994). This plant possesses a gametophytic self-incompatibility system (GSI) (i.e., phenotypic recognition determined by a haploid gametophyte; Pandey, 1957) controlled by an S-locus (Mulato-Brito et al., 2007). The GSI system is characterized by late recognition of self-pollen tubes at the pistil. Pollen grains placed on stigmas by self-pollination germinate but the growth of pollen tubes is limited to the first third of pistil (Richards, 1997; Carvalho et al., 2020). It is known that the common ancestor of *Physalis* and *Witheringia* suffered a historical bottleneck that reduced S-allele diversity in three S-lineages, and its subsequent rapid diversification resulted in current species with a number of alleles similar to other Solanaceae genera (Igic et al., 2003).

Although *P. philadelphica* has been categorized as a self-incompatible species, it is possible to find some self-compatible individuals in husk tomato crops and wild populations (Pandey, 1957; Labate and Robertson, 2015). This may be attributed to a mutation in the S locus (Mulato-Brito et al., 2007). Thus, *P. philadelphica* is considered a partially self-incompatible species, and it has wide morphological variation in flowers and fruits between wild and cultivated plants (Hudson, 1986; Montes-Hernández and Aguirre-Rivera, 1994). This species presents an evolutionary gradient from wild plants, landraces, and crops (Zamora-Tavares et al., 2015) as well as different reproductive strategies. Consequently, *P. philadelphica* makes a suitable model for the study of changes in reproductive strategies associated with different degrees of domestication.

This study aimed to investigate changes in the reproductive system and parameters in wild (weedy plants), semi-domesticated (landraces), and domesticated plants (commercial plants) of *P. philadelphica*. We hypothesized that artificial selection favors self-compatibility in cultivated plants by changing their reproductive strategy and some reproductive parameters associated with domestication. To test this hypothesis, we first characterized their floral morphology and reproductive parameters of weeds, landraces, and commercial plants. Next, we conducted an artificial crossing experiment among plants with different degrees of domestication. Then, the seeds obtained from this experiment were germinated and seedling growth was recorded.

## Materials and Methods

### Biological Material

In this study, we classified plants of different degrees of domestication considering the habitat in which they grow, namely natural or crop areas, as well as morphometric and genetic characteristics as classification criteria. We classified plants as weedy plants, landraces, and commercial plants. Weedy wild plants are those which grow spontaneously in forest or milpa agroecosystems (i.e., agroecosystems where corn, beans and squash planted in association). They produce small fruits (<2 cm) and flowers (1.2–1.9 cm of corolla diameter). Natural populations of weedy plants are not subjected to artificial selection (non-domesticated populations), but they sometimes grow in association with humans in disturbed habitats (i.e., ruderals). People usually pick their fruits to sell them in local markets or for home consumption (Pre-Stage 1) (Zamora-Tavares et al., 2015). In the context of our research, we referred to them as *weedy plants* (code OVP337, OVP274, PZ275B, and SN). Landraces are cultivated plants with a historical origin (tradition varieties), which have been locally adapted and associated with traditional farming. They have a distinct identity and are genetically diverse, but lack formal crop improvement (Camacho-Villa et al., 2005). These traditional plants are artificially selected and belong to semi-domesticated populations (Stage 2). We recorded the fruits of landraces as medium size (2.5–3.5 cm) and with different colors (yellow, green, and purple). They are referred to in our study as landraces (code OVP2007 and JS522). Commercial plants were also used as biological materials; their seeds were obtained from commercial companies (code JS1, JS7, JS35, and JS347). The fruits of commercial plants were the largest (>3.5 cm). The green fruits are usually the most popular ones and are marketed on a larger scale in the country. The purple or green ones with purple hues are rather commercialized in the western region of Mexico (domesticated populations or Stage 3). Only JS347, which is a hybrid obtained by the Universidad Autónoma Chapingo, was considered to be in Stage 4. Herbarium specimens were deposited in ECO-TAP (Table 1).

**TABLE 1:** Characteristics of flowers (corolla diameter range) and fruits (color and size), and origin of seeds germinated in greenhouse for description of reproductive systems.

**Code**	**Degree of domestication**	**Name**	**Company/Population**	**Corolla diameter of flowers (cm)**	**Color and size (cm) of fruits**	**Herbarium code**
JS1	Commercial	Morada Plus	Optimus seed	1.8–2.7	Purple fruit/large size (3.5–5.0)	HET 1925
JS7	Commercial	Morada R		1.7–2.7	Purple fruit/large size (3.5–4.5)	HET 1926
JS35	Commercial	Corral Blanco		1.6–2.6	Green fruit/large size (3.5–5.0)	
JS347	Commercial	Esmeralda	Chapingo, San Miguel Allende Guanajuato	1.0–3.1	Green fruit/large size (3.5–6.0)	HET 1929
OVP2007	Landrace	Morado Molina	Cuquío, Jalisco	1.8–2.6	Purple fruits/medium size (2.5–3.5)	
JS522	Landrace		Texmelucan, Puebla	1.4–2.4	Yellow and green fruit/medium size (2.5–3.5)	HET 1927
OVP337	Wild/Weedy		Yahualica, Jalisco		Green and purple fruit/small size (1.2–2.0)	
OVP274	Wild/Weedy		Altamirano, Guerrero	1.4–1.8	Green and purple fruit/small size (1.2–2.0)	
SN	Wild/Weedy		Altamirano, Tenejapa, Chiapas	1.2–1.9	Green fruit/small size (1.2–2.0)	HET 1928

*Physalis philadelphica* seeds were collected at different places from 2009 to 2012 or were bought from different companies in 2009 (Table 1). When we collected weedy plants, depending on population size, we picked some fruits from 20 to 50 individuals to produce a homogenous and representative sample. All fruits collected in one site were placed in a paper bag to transport to the laboratory. Later seeds were obtained and mixed to make a homogenous sample. In the case of the landraces, we sampled 30 plants of each traditional crop, taking care that they were in different rows and separated from each other. From each plant, we harvested one fruit and mixed them to make up the representative sample of each site. Finally, seeds from commercial plants were bought from different companies as described in Table 1. Seeds were stored at 4°C until planting.

### Plant Growth

To obtain plants to describe the modification in reproductive systems during the domestication process, we germinated seeds of plants with different degrees of domestication: commercial plants (JS1, JS7, JS35, and JS347), landraces (OVP2007 and JS522), and weedy plants (OVP337, OVP274, PZ275B, and SN). We sowed 10 to 20 seeds per section at three times: January 2016, August 2016, and December 2017. The seeds were previously soaked in tap water for 2 h and, subsequently, sowed in germination trays in the greenhouse at El Colegio de la Frontera Sur (ECOSUR; 14° 53′ 194″ N, 92° 17′ 18″ W, 152 masl), Tapachula, Chiapas, Mexico. After 2 weeks, plants were transplanted into pots (5 cm diameter) and, after four more weeks, they were transplanted into larger pots (15 cm of diameter) with slow-release fertilizer (NUTRI-TABS, Miracle-Gro, Netherland) until they began to bloom. We conducted the experiments described below and measured floral traits during three periods: February to April 2016, September to November 2016, and January to March 2017.

### Floral Morphology Variation Between Weedy and Cultivated Plants

This experiment was performed to determine the variation in floral morphology during the domestication process. We measured 2–3 flowers from 5 to 7 individuals per accession (a total of 134 flowers from 46 individuals) from September to November 2016. We measured the following 15 traits using a digital caliper (Steren) when flowers were completely open: (1) length (LS) and (2) width of one sepal (WS); (3) corolla diameter (CD); (4) length (LA) and (5) width of one anther (WA); (6) length (LF) and (7) width of the anther filament (WF); (8) length (LS) and (9) width of style (WS); (10) stigmatic area (STA); (11) length (LO) and (12) width of ovary (WO); (13) length (LN) and (14) width of nectary guides (WN); and (15) distance between anthers and stigma (herkogamy). In addition, we measured the size of 5 pollen grains per flower.

### Pollen: Ovule Ratio Between Weedy and Cultivated Plants

In this experiment, we calculated the pollen: ovule ratio (i.e., the ratio of the amount of pollen to the number of ovules in the flower; Erbar and Langlotz, 2005). We counted the number of ovules from the same flowers used in the floral morphology section and the number of pollen grains from anthers previously collected to dehiscence during the second and third season (6–11 flowers per accession). We placed one anther per flower in a 1 ml tube with a pinch of powdered detergent (Salvo, Mexico). This was then macerated, and the anther tissue was eliminated (Márquez-Guzmán et al., 2016). We counted the number of pollen grains with a Newbauer chamber using a microscope (AxioLab A1, Zeiss, Germany). Counting was performed four times per sample. We obtained an average per sample to extrapolate for the initial volume. Later, we multiplied this number by five anthers to obtain the total amount of pollen per flower.

### Artificial Crossing Experiment

We conducted experimental manipulation in a greenhouse, which excluded plants from pollinators, to investigate change in the *P. philadelphica* reproductive system. As flowers opened (i.e., corolla was completely expanded usually within 2 days after anthesis) and the style extended its wet stigma, the following treatments were applied from 9:00 to 14:00 h following Eckert et al. (2010). Some modifications were made as further described. (1) Emasculation (E). The anthers were removed before dehiscence at the floral-bud stage or on the first day of anthesis. This was used to determine whether different accessions of *P. philadelphica* set seed without pollination (i.e., through agamospermy). (2) Pollinator exclusion (PE). Flowers were left intact until senescence. The purpose of this treatment was to assess the plant’s ability to self-fertilize in the absence of pollinators (i.e., autonomous autogamy). To quantify the pollen deposited autonomously on the stigma of the flowers previously used for morphological measurements, the terminal end of the style, together with the stigma, was cut and placed on a slide with fuchsine-stained gel (Kearns and Inouye, 1993). (3) Hand-mediated self-pollination (S). The pollen, from the same flower, was deposited, on its stigma. The purpose of doing this was to assess the plant’s ability to accept or reject its own pollen grains (i.e., self-compatibility system). (4) Hand-mediated outcross pollination (CP). Pollen used in this treatment was gathered from three individuals from a different accession. The flowers that received this pollen were previously emasculated. In hand-mediated pollination, pollen was deposited on receptive stigmas to maximize grain deposition. Specifically, the white pollen was added until they were visible to the naked eye. Commercial, landrace, and weedy accessions were inter-crossed so individuals of each accession were pollen donors (male parent) and recipients (female parent). Each treatment was carried out in duplicate on 15 individuals per accession in each season.

After the artificial crossing experiment had been carried out, we quantified the fruit set. Subsequently, we weighed and measured (length and width) the fruits. Later, we quantified the seed sets by distinguishing between viable and aborted seeds. We weighed only the viable seeds, and sowed 50 seeds per treatment (in total 550 seeds) following methods previously described.

### Seeds Germination and Seedling Growth

We recorded the day the seeds germinated and calculated the germination rate during 15 days of observation. Moreover, we recorded the day after sowing (DAS) that cotyledons and the embryonic foliage leaves emerged (Valdivia-Mares et al., 2016). In addition, we measured seedling height when cotyledons emerged (i.e., from base of seedling to the point of cotyledons emergence) and one week after germination. We measured the length of one cotyledon a week after germination and the length of one embryonic foliage leaf five days after their emergence (i.e., from cotyledon or leaf base to apex). Finally, we calculated the proportion of seedlings that survived 23 days after sowing.

### Statistical Analysis

For the analyses, we used R statistical software, ver. 3.6.0 (R Core Development Team, 2019). We conducted a principal components analysis (PCA) with floral trait measurements to establish whether flower size differed in *P. philadelphica* according to domestication degree. We used seven separate linear mixed models (LMM) to analyze the difference between the reproductive parameters measured in the three degrees of domestication commercial, landrace, and weedy plants as shown in Supplementary Table 1. Each model used the degree of domestication as a fixed effect, with plant identity and population origin as random effects. Random effects that were not significant were eliminated from each model. The variable used as a fixed effect was log-transformed. For each model, we conducted an analysis of variance (ANOVA) to determine statistical differences and the Tukey test using the glht function from the multcomp library (Hothorn et al., 2008). Mixed models were developed using the lmerTest package (Zeileis and Hothorn, 2002). The variance and covariance of the random effects were obtained using the ranef function (package lme4).

In addition, we conducted 17 separate generalized linear models (GLM) to analyze the difference in fruit set, seed set, and percentage of germination among treatments. We fit fruit set and percentage of germination with binomial error and seed set with Poisson error. Treatments without fruit production were excluded. In addition, we calculated the confidence intervals using the Hmisc package to compare the fruit sets between the emasculation and the self-pollination treatments (Harrell and Dupont, 2019). We conducted 12 separate GLM analyses fitted with Gaussian error to analyze the difference in fruit weight, length, width, and viable seed weight among treatments of artificial crosses. Afterward, we conducted multiple comparisons of means (Tukey contrasts) using the glht function from multcomp library. We conducted a survival analysis (Kaplan-Meier method) using the survival package (Therneau and Grambsch, 2000) in order to analyze whether the probability of germination differed among treatments over 15 days of observation as well as the probability of seedling survival among treatments over 23 days of observation. To graph, we used the survival probability using the survminer package (Alboukadel and Kosinski, 2019). In the case of the germination graph, we used the inverse of survival probability. We used 10 separate GLM fit with Poisson error and Tukey test to compare time of emergence of cotyledons and the embryonic foliage leaves. Finally, we used six separate GLM fit with Gaussian error to analyze the difference in plant height at the time cotyledons emerged and one week after germination. We used another six separate GLM fit with Gaussian error to analyze the difference in cotyledon and embryonic foliage leaf size.

## Results

### Floral Morphology Variation Between Weedy and Cultivated Plants

**FIGURE 1:**
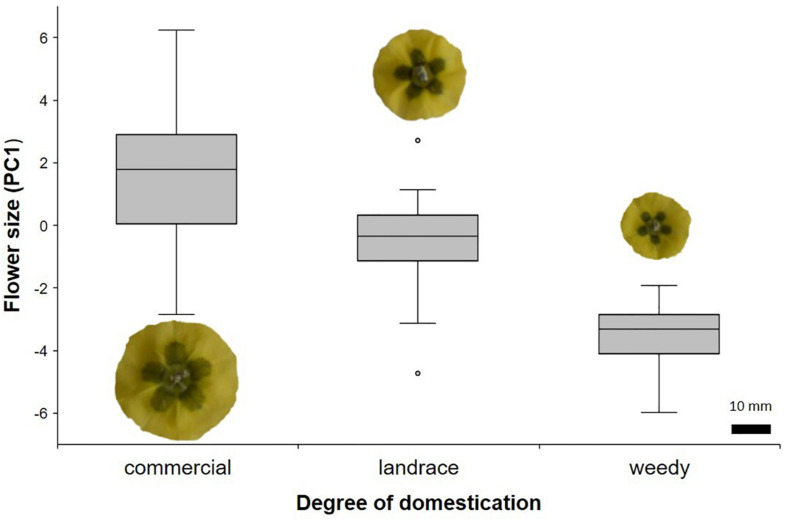
Flower size among different degrees of domestication in *Physalis philadelphica* represented by scores of the first principal component.

The PCA showed that the first component (PC1) could explain 57.3% of the variance in floral morphology. Size was represented by this component as all eigenvalues have similar signs and values (Supplementary Table 2). The ANOVA with the PC1 scores showed that commercial individuals had the largest flowers, while weedy individuals had the smallest ones (*F*_2_,_131_ = 92.04, *P* < 0.001). Landraces had intermediate size flowers compared with the other two degrees of domestication (Figure 1). No differences in other reproductive parameters were found (Table 2), except for the number of ovules, which was found to be higher in commercial plants than in landraces. Weedy plants had the lowest number of ovules (Table 2; Supplementary Table 1).

**TABLE 2:** Reproductive parameters in *Physalis philadelphica* with different degrees of domestication.

**Code**	**Degree of domestication**	**Stigmatic area (mm^2^)**	**Pollen grain size (μm)**	**Distance stigma-anthers (mm)**	**No. pollen grains deposited on stigma**	**No. ovules**	**No. pollen grains per flower (×10^3^)**	**Pollen:ovule proportion**
JS1	Commercial	0.22 ± 0.02 (24)	22.1 ± 0.5 (120)	0.6 ± 0.1 (24)	145.9 ± 8.7	179.4 ± 6.2	149 ± 25 (11)	864 ± 45.0
JS7	Commercial	0.21 ± 0.04 (21)	22.4 ± 0.3 (105)	0.9 ± 0.1 (21)	134.0 ± 10.1	155.7 ± 7.3	141 ± 23 (7)	949 ± 43.1
JS35	Commercial	0.22 ± 0.03 (14)	20.4 ± 0.3 (70)	1.1 ± 0.1 (14)	113.4 ± 16.2	134.6 ± 14.0	132 ± 63 (3)	1120 ± 102.9
JS347	Commercial	0.45 ± 0.05 (15)	24.0 ± 0.5 (75)	0.4 ± 0.2 (15)	190.5 ± 13.7	204.5 ± 7.7	236 ± 39 (6)	1180 ± 47.4
Mean		0.26 ± 0.02	21.9 ± 0.2	0.7 ± 0.08	145.4 ± 6.4	169.3 ± 4.5a	164 ± 17	1000.6 ± 31.9
OVP2007	Landrace	0.17 ± 0.02 (17)	20.9 ± 0.4 (85)	0.6 ± 0.1 (17)	100.2 ± 8.2	94.9 ± 2.3	244 ± 33 (9)	2592 ± 62.7
JS522	Landrace	0.19 ± 0.02 (14)	20.3 ± 0.4 (70)	1.6 ± 0.3 (14)	101.3 ± 12.7	94.7 ± 4.6	129 ± 26 (7)	1412 ± 70.8
Mean		0.18 ± 0.01	20.6 ± 0.3	1.0 ± 0.17	100.7 ± 7.2	94.8 ± 2.4b	194 ± 26	2059.2 ± 116.8
OVP274	Weedy	0.59 ± 0.08 (15)	22.4 ± 0.4 (75)	0.8 ± 0.2 (15)	149.7 ± 14.0	66.3 ± 3.3	76 ± 9 (7)	1212 ± 83.3
SN	Weedy	0.09 ± 0.02 (14)	19.8 ± 0.6 (70)	0.5 ± 0.1 (14)	53.7 ± 6.6	46.1 ± 3.9	113 ± 21 (7)	2673 ± 218.1
Mean		0.34 ± 0.06	21.2 ± 0.4	0.6 ± 0.13	103.3 ± 11.9	56.5 ± 3.13c	95 ± 12	1917.5 ± 177.5

*In parenthesis the number of repetitions: stigma-anther distance, number of pollen grains deposited on stigma, number of ovules and pollen:ovule ratios have the same number of repetitions. The statistical analyses are described in detail in Supplementary Information 1. Different letters indicate statistical differences between degrees of domestication.*

### Artificial Crossing Experiment

*Physalis philadelphica* did not produce fruits through agamospermy (Table 3). Landraces of this species did not develop fruits from hand-mediated self-pollinated flowers. In the same treatment, weedy and commercial plants produced very few fruit sets (0.8% each treatment). These results were not different from those obtained in the emasculation treatment CI commercial plants (0–0.06) and in hand-mediated self-pollination (0.008–0.003). They did not differ from the emasculation treatment CI weedy plants (0–0.28) and hand-mediated self-pollination (0.0004–0.04). Conversely, we found that all degrees of domestication produced fruits when they were excluded from pollinators. Weedy plants produced the highest fruit set (2.4:1, ratio of SC to SI individuals), followed by landraces (1:1.3) and commercial plants (1: 8.2). These fruits, which represent all degrees of domestication, were the smallest and the lightest (Table 4). The outcross pollination treatments differed significantly when commercial plants functioned as pollen receptors and landraces or weedy plants functioned as pollen donors. The highest percentage of fructification was obtained when weedy plants donated pollen to commercial plants (Table 3).

**TABLE 3:** Fruit set (FS; percentage of flowers maturing into fruits) and mean seed set (SS; percentage of viable seeds) of artificial crosses in *Physalis philadelphica* with different degrees of domestication.

**Treatments**	**Commercial plants**		**Landraces**		**Weedy plants**	
	**Fruit set**	**Seed set (No. seeds)**	**Fruit set**	**Seed set (No. seeds)**	**Fruit set**	**Seed set (No. seeds)**
Emasculation	0 (62)	0	0 (15)	0	0 (10)	0
Pollinator exclusion	11 (358)b,A	84 ± 3.8c (9 ± 2.2a)	43 (56)B	80 ± 4.3 (18 ± 4.9a)	72 (60)c,C	92 ± 2.2 b (33 ± 3.5a)
Hand-mediated self-pollination	0.8 (497)a	61 ± 12.03a (62 ± 21.2b)	0 (298)	0	0.8 (127)a	74ab (76c)
Commercial vs. Landrace	20 (73)b	75 ± 6.8b (81 ± 16.7c)				
Commercial vs. Weedy	48 (71)c	81 ± 4.2bc (101 ± 11.4d)				
Landrace vs. Commercial			20 (30)	81 ± 15.8 (92 ± 31.5c)		
Landrace vs. Weedy			42 (36)	84 ± 4.8 (77 ± 11.4b)		
Weedy vs. Commercial					20 (39)b	81 ± 5.4a (51 ± 12.1b)
Weedy vs. Landrace					24 (21)b	77 ± 15.4a (31 ± 9.1a)
	FS: Deviance = 148.58, DF= 3***		FS: Deviance = 5.223, DF= 2^*NS*^		FS: Deviance = 121.02, DF= 3***	
	SS: Deviance = 30.84, DF= 3***		SS: Deviance = 1.132, DF= 2^*NS*^		SS: Deviance = 20.533, DF= 3***	
	NS: Deviance = 3401.3, DF= 3***		NS: Deviance = 918, DF= 2***		NS: Deviance = 90.383, DF= 3***	
					PE: Deviance = 109.03, DF= 2***	

In parenthesis, number of flowers or mean number of viable seeds (NS) ± standard error. Different lower case letters indicate statistical differences between treatments in each degree of domestication. Different upper case letters indicate statistical differences between degrees of domestication in each treatment. ^*NS*^*P* > 0.05, *P < 0.05, **P < 0.01, and ***P < 0.001.

**TABLE 4:** Mean fruit weight, length and width and mean weight of viable seeds (±standard error).

**Treatments**	**Fruit weight (g)**	**Fruit length (mm)**	**Fruit width (mm)**	**Seed weight (g)**
**Commercial plants**				
Pollinator exclusion	1.97 ± 0.26a	13.80 ± 0.68a	14.62 ± 0.74a	0.0018 ± 0.0001b
Hand-mediated self-pollination	8.11 ± 2.08b	24.09 ± 2.05b	24.64 ± 2.30b	0.0014 ± 0.0002ab
Commercial vs. landrace	7.48 ± 2.02b	20.16 ± 1.76b	23.63 ± 1.83b	0.0015 ± 0.00004ab
Commercial vs. weedy	7.94 ± 0.81b	21.94 ± 0.89b	24.43 ± 1.02b	0.0013 ± 0.00008a
*Statistical test*	Deviance = 752, DF = 3, *P* < 0.001	Deviance = 1396, DF = 3, *P* < 0.001	Deviance = 2025, DF = 3, *P* < 0.001	Deviance = 5 × 10^–6^, DF = 3, *P* < 0.01
**Landraces**				
Pollinator exclusion	1.64 ± 0.22a	13.27 ± 0.80a	13.83 ± 0.84a	0.0019 ± 0.001b
Landrace vs. commercial	3.42 ± 0.55b	17.00 ± 0.74b	19.70 ± 1.23b	0.0012 ± 0.0002a
Landrace vs. weedy	3.13 ± 0.44b	16.79 ± 0.62b	17.81 ± 0.83b	0.0013 ± 0.0002a
*Statistical test*	Deviance = 26.8, DF = 2, *P* < 0.001	Deviance = 136.7, DF = 2, *P* < 0.01	Deviance = 237.0, DF = 2, *P* < 0.001	Deviance = 3 × 10^–6^, DF = 2, *P* < 0.01
**Weedy plants**				
Pollinator exclusion	0.50 ± 0.04a	9.50 ± 0.31a	9.48 ± 0.36a	0.0012 ± 0.00004
Hand mediated self-pollination	1.10ab	12.44ab	12.51ab	0.0011
Weedy vs. commercial	1.63 ± 0.67b	12.19 ± 1.76b	12.72 ± 2.25b	0.0011 ± 0.00007
Weedy vs. landrace	0.68 ± 0.16ab	10.74 ± 0.98ab	10.47 ± 0.92ab	0.0012 ± 0.00015
*Statistical test*	Deviance = 8.7, DF = 3, *P* < 0.01	Deviance = 57.14, DF = 3, *P* = 0.04	Deviance = 77.02, DF = 3, *P* = 0.05	Deviance = 3 × 10^–6^, DF = 3, *P* = 0.87

*Different lower case letters indicate statistical differences among treatments in each degree of domestication.*

In commercial plants, a significant difference in seed set was found between the seed sets from the pollinator exclusion treatment and those from the outcross pollination treatment when landraces donated pollen. These treatments resulted in the highest and the lowest values, respectively (Table 3). As we expected, the lowest numbers of viable seeds were produced in the pollination exclusion and hand-mediated self-pollination treatments. In contrast, the highest number of seeds was found in the outcross treatment when weedy plants donated pollen (*n* = 101 viable seeds). With regard to landraces, there were no differences in seed sets among treatments (Table 3). The highest number of viable seeds was obtained between landraces and commercial plants (92) in the outcrossed treatment. The lowest number was obtained in the pollination exclusion treatment (18). This treatment produced the heaviest seeds. Finally, in weedy plants the seed set did not differ between outcross pollination treatments, but it was higher in the pollination exclusion treatment. The lowest number of viable seeds was found in the pollination exclusion (33) and outcross treatments (31) when landraces functioned as pollen donors. The highest number of viable seeds was found in the self-pollination treatment (76).

### Germination

**FIGURE 2:**
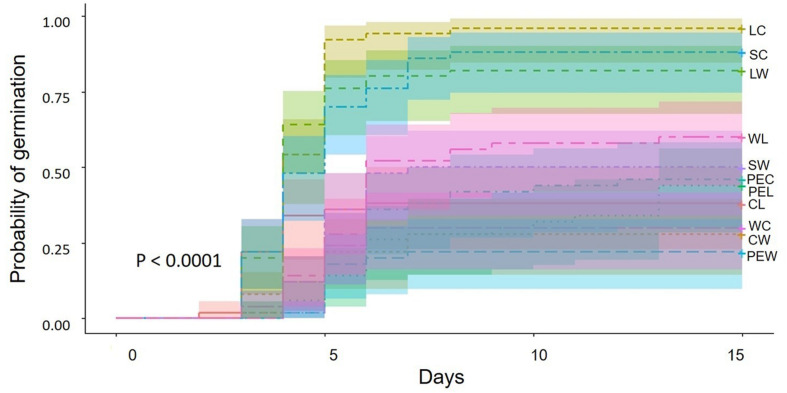
Germination probability and intervals of confidence of seeds produced by artificial crossing of *Physalis philadelphica* during 15 days of observation. Abbreviations: LC (landraces vs. commercial plants), SC (self-pollination in commercial plants), LW (landraces vs. weedy plants), WL (weedy plants vs. landraces), SW (self-pollination in weedy plants), PEC (pollinator exclusion in commercial plants), PEL (pollinator exclusion in landraces), CL (commercial plants vs. landraces), WC (weedy vs. commercial plants), CW (commercial vs. weedy plants), PEW (pollinator exclusion in weedy plants).

The seeds resulting from outcrossing between landraces and commercial plants showed the highest germination percentage, followed by the hand mediated self-pollination treatment in commercial plants and outcrossing between landraces and weedy plants (Table 5). In these three treatments, most of the seeds germinated between day 4 and 5 (Figure 2). Conversely, seeds produced by spontaneous self-pollination in weedy plants had the lowest germination percentage, followed by outcrossing between commercial and weedy plants and the reciprocal cross. Seeds of spontaneous self-pollination and commercial and weedy crosses germinated on day 3 and 7 after sowing (Figure 2).

**TABLE 5:** Percentage of germination of artificial crosses in *Physalis philadelphica* with different degrees of domestication.

	**Commercial plants (C)**		**Landraces (L)**		**Weedy plants (W)**	
**Treatments**	**Percentage (%)**	**Days after sowing (DAS)**	**Percentage (%)**	**Days after sowing (DAS)**	**Percentage (%)**	**Days after sowing (DAS)**
Pollinator exclusion (PE)	44 a	7.68 ± 0.74b,C	46a	5.78 ± 0.44b,AB	22a	5.00 ± 0.15A
Hand-mediated self-pollination (S)	88b	4.51 ± 0.20a			50bc	5.21 ± 0.10
Commercial vs. Landrace (CL)	38a	3.53 ± 0.21a				
Commercial vs. Weedy (CW)	28a	4.71 ± 0.40a				
Landrace vs. Commercial (LC)			96b	4.5 ± 0.11a		
Landrace vs. Weedy (LW)			82b	4.10 ± 0.16a		
Weedy vs. Commercial (WC)					30ab	4.67 ± 0.25
Weedy vs. Landrace (WL)					60c	5.76 ± 0.35
	% Deviance = 46.2, DF = 3***		% Deviance = 36.9, DF = 2***		%Deviance = 19.6, DF = 3***	
	DAS Deviance = 38.63, DF = 3***		DAS Deviance = 8.8, DF = 2*		DAS Deviance = 2.509, DF = 3^*NS*^	
					PE Deviance = 10.03, DF = 2**	

Different lower case letters indicate statistical differences between treatments in each degree of domestication. Different upper case letters indicate statistical differences between degrees of domestication in each treatment. ^*NS*^*P* > 0.05, *P < 0.05, **P < 0.01, and ***P < 0.001.

### Emergence of Cotyledons and Embryonic Foliage Leaves

In plants resulting from the commercial plant pollinator exclusion treatment, the cotyledons and the embryonic foliage leaves emerged later than those in weedy plants (Table 6). However, cotyledon emergence in landraces was not different from that in commercial and weedy plants. Emergence of the embryonic foliage leaves differed only in commercial plants.

**TABLE 6:** Mean days after sowing (DAS) of cotyledon emergence and mean DAS of appearance of embryonic foliage leaves (±standard error) of artificial crosses in *Physalis philadelphica* with different degrees of domestication.

	**Commercial plants (C)**		**Landraces (L)**		**Weedy plants (W)**	
**Treatment**	**Cotyledons (Co)**	**Embryonic foliage leaves (EFL)**	**Cotyledons (Co)**	**Embryonic foliage leaves (EFL)**	**Cotyledons (Co)**	**Embryonic foliage leaves (EFL)**
Pollinator exclusion (PE)	8.8 ± 0.77b,B	10.6 ± 0.87b,C	6.9 ± 0.48b, AB	8.4 ± 0.53AB	6.0 ± 0.15A	7.6 ± 0.27A
Hand-mediated self-pollination (S)	5.6 ± 0.23a	7.6 ± 0.19a			6.2 ± 0.10	8.4 ± 0.12
Commercial vs. Landrace (CL)	4.7 ± 0.33a	6.4 ± 0.32a				
Commercial vs. Weedy (CW)	5.6 ± 0.40a	7.3 ± 0.33a				
Landrace vs. Commercial (LC)			5.7 ± 0.17ab	7.7 ± 0.16		
Landrace vs. Weedy (LW)			5.1 ± 0.16a	7.2 ± 0.15		
Weedy vs. Commercial (WC)					6.3 ± 0.44	8.5 ± 0.39
Weedy vs. Landrace (WL)					6.9 ± 0.42	8.4 ± 0.42
	CoC: Deviance = 31.69, DF= 3***		CoL: Deviance = 8.16, DF= 2*		CoW: Deviance = 1.50, DF= 3^*NS*^	
	EFLC: Deviance = 25.24, DF= 3***		EFLL: Deviance = 2.28, DF= 2^*NS*^		EFLW: Deviance = 0.79, DF= 3^*NS*^	
	CoPE: Deviance = 8.81, DF= 2**		CoS: Deviance = 0.95, DF=1^*NS*^			
	EFLPE: Deviance = 9.06, DF= 2**		EFLS: Deviance = 1.19, DF= 1^*NS*^			

Different lower case letters indicate statistical differences between treatments in each degree of domestication. Different upper case letters indicate statistical differences between degrees of domestication in each treatment. ^*NS*^*P* > 0.05, *P < 0.05, **P < 0.01, and ***P < 0.001.

When comparing all treatments in the same degree of domestication, it was found that, in commercial plants, the cotyledons and the embryonic foliage leaves of the pollinator exclusion treatment emerged later than those of the other treatments. With regard to landraces, there were differences in cotyledon emergence. Cotyledons of plants resulting from the pollinator exclusion treatment emerged later than those from outcrossing between landraces and weedy plants. However, they did not differ in the crosses between landraces and commercial plants treatments. Finally, emergence of cotyledons and embryonic foliage leaves did not differ among other treatments in weedy plants.

### Seedling Growth

When cotyledons emerged, seedling height did not differ among treatments in commercial plants (Table 7), but differences in seedling height were observed in landraces and weedy plants. In landraces, the seedlings resulting from the pollen exclusion treatment and those from the cross between landraces and weedy plants were taller than those of the cross between landraces and commercial plants. Weedy seedlings resulting from the hand-mediated self-pollination treatment were shorter than those in all of the other treatments.

**TABLE 7:** Mean seedlings height when cotyledons emerged (SHCE) and a week after germination (SHWAG) (±standard error) resulting from different treatments of artificial crosses experiment.

	**Commercial plants (C)**		**Landraces (L)**		**Weedy plants (W)**	
**Treatments**	**Seedling height when cotyledons emerged (cm)**	**Seedling height a week after germination (cm)**	**Seedling height when cotyledons emerged (cm)**	**Seedling height a week after germination (cm)**	**Seedling height when cotyledons emerged (cm)**	**Seedling height a week after germination (cm)**
Pollinator exclusion (PE)	1.47 ± 0.11	4.50 ± 0.44c	1.70 ± 0.08a	5.03 ± 0.36b	1.47 ± 0.09a	3.6 ± 0.33b
Hand-mediated self-pollination (S)	1.75 ± 0.11	6.28 ± 0.32b			1.15 ± 0.05b	2.71 ± 0.08b
Commercial vs. Landrace (CL)	1.79 ± 0.15	7.37 ± 0.34ab				
Commercial vs. Weedy (CW)	2.08 ± 0.25	5.49 ± 0.66abc				
Landrace vs. Commercial (LC)			1.06 ± 0.04b	4.98 ± 0.11b		
Landrace vs. Weedy (LW)			1.46 ± 0.09a	6.00 ± 0.31a		
Weedy vs. Commercial (WC)					1.23 ± 0.12a	5.73 ± 0.39a
Weedy vs. Landrace (WL)					1.47 ± 0.08a	5.59 ± 0.27a
	SHCEC: Deviance = 3.22, DF= 3^*NS*^		SHCEL: Deviance = 7.13, DF= 2***		SHCEW: Deviance = 1.73, DF= 3**	
	SHWAGC: Deviance = 92.26, DF= 3***		SHWAGC: Deviance = 25.45, DF= 2**		SHWAGW: Deviance = 143.24, DF= 3***	

Different lower case letters indicate statistical differences between treatments in each degree of domestication. ^*NS*^*P* > 0.05, *P < 0.05, **P < 0.01, and ***P < 0.001.

Although this pattern changed, when we measured seedling height one week after germination, seedlings resulting from autonomous autogamy in commercial plants were the shortest. They did not differ from those plants resulting from crosses commercial and weedy plants (Table 7). The seedlings resulting from crosses landraces and weedy plants were taller than landraces used in the other treatments. Finally, seedlings resulting from the crosses between weedy vs. commercial plants and weedy plants vs. landraces were almost double in size compared to seedlings from the autogamy treatments (pollination exclusion and hand-mediated self-pollination).

Cotyledon and embryonic foliage leaf size (length) of seedlings resulting from autogamy were smaller than those of outcrosses between commercial plants and landraces and their reciprocal cross (Table 8). However, in both variables weedy seedlings resulting from autogamy were not different from those in outcrosses. Finally, there was no significant difference in the probability of seedling survival (*P* = 0.57) among treatments over 23 days of observation (93–100% of the seedlings survived; Table 8).

**TABLE 8:** Mean left cotyledon length a week after germination and left embryonic foliage leaf length 5 days after emergence (±standard error) resulting from different treatments of the artificial crossing experiment.

	**Commercial plants (C)**		**Landraces (L)**		**Weedy plants (W)**	
**Treatments**	**One cotyledon length (cm)**	**One embryonic foliage leaf length (cm)**	**One cotyledon length (cm)**	**One embryonic foliage leaf length (cm)**	**One cotyledon length (cm)**	**One embryonic foliage leaf length (cm)**
Pollinator exclusion (PE)	1.02 ± 0.07b	0.92 ± 0.11b (100%)	1.14 ± 0.10b	1.20 ± 0.11b (95.7%)	1.09 ± 0.04	1.37 ± 0.10 (99.9%)
Hand-mediated self-pollination (S)	1.07 ± 0.05b	1.48 ± 0.10b (93.2%)			1.08 ± 0.02	2.10 ± 0.07 (96%)
Commercial vs. Landrace (CL)	1.34 ± 0.03a	1.86 ± 0.18a (94.7%)				
Commercial vs. Weedy (CW)	1.20 ± 0.04ab	1.61 ± 0.15ab (92.9%)				
Landrace vs. Commercial (LC)			1.33 ± 0.02a	2.06 ± 0.07a (100%)		
Landrace vs. Weedy (LW)			1.28 ± 0.05ab	1.73 ± 0.09a (97.6%)		
Weedy vs. Commercial (WC)					1.11 ± 0.05	1.19 ± 0.06 (100%)
Weedy vs. Landrace (WL)					1.14 ± 0.05	1.35 ± 0.08 (100%)
	Cotyledon: Deviance = 1.33, DF= 3***		Cotyledon: Deviance = 0.55, DF= 2*		Cotyledon: Deviance = 0.05, DF= 3^*NS*^	
	Leaf: Deviance = 3.27, DF= 3***		Leaf: Deviance = 1.68, DF= 2***		Leaf: Deviance = 0.56, DF= 3^*NS*^	

Different lower case letters indicate statistical differences between treatments in each degree of domestication. In parenthesis, the proportion of seedlings that survived 23 days after sowing. ^*NS*^*P* > 0.05, *P < 0.05, **P < 0.01, and ***P < 0.001.

## Discussion

Since the domestication process prefers self- to cross-fertilization, artificial selection usually suppresses or removes self-incompatibility and outcrossing traits (Dempewolf et al., 2012). Contrary to our expectations, by comparing other domesticated species in the Solanaceae, we found that in *P. philadelphica* artificial selection favors SI over SC. Weedy plants presented higher fruit set produced by autonomous autogamy compared to cultivated plants. Spontaneous self-pollination seems to be advantageous in weedy populations because it produces viable seeds, with cotyledons and embryonic foliage leaves emerging before those of cultivated plants. These changes in the reproductive strategy are also associated with increments in flower size and ovule production indicative to the domesticated syndrome.

### Changes in Reproductive Strategy During Domestication

The domestication syndrome is defined as crops having traits that diverge from wild relatives, including those associated with changes in reproductive strategies (Meyer et al., 2012). In this study, we found that cultivated plants of *P. philadelphica* differ from weedy plants in flower size and ovule numbers. Reduction in floral size is related to a reduction in distance between anthers and stigma, usually associated with an increase in self-fertilization. For example, in six SC species of *Solanum* section *Androceras* transition from large to small flowers and lower P:O ratios suggest a transition toward self-fertilization (Vallejo-Marín et al., 2014). It seems that the breakdown of SI is followed by a rapid evolution of selfing attributes, such as a reduction in flower size (Igic et al., 2008) and floral longevity (Broz et al., 2017). For example, it has been reported that some SC species of *Solanum* sect. *Lycopersicon* show decreased flower size and stigma exertion (Rick, 1988; Bedinger et al., 2010). Regarding the number of ovules, commercial *P. philadelphica* plants produce three times more ovules than weedy plants. However, weedy plants are proportionally more efficient in seed production considering the lower number of ovules. Similar results have been reported in cotton, *Gossypium hirsutum*, since commercial plants present twice as many ovules as wild plants despite producing a proportionally smaller number of seeds under natural pollination conditions (Velázquez-López et al., 2018).

In the artificial crossing experiment, our results suggest that *P. philadelphica* is a partially self-incompatible species and that domestication selects for SI in cultivars. Previously, Mulato-Brito et al. (2007) suggested that the self-incompatibility (SI) system is the common condition in cultivated plants, while the self-compatible (SC) system is common in wild plants of *P. philadelphica*. In this study, we confirmed this assumption. We found that *P. philadelphica*, in all degrees of domestication, could produce fruits autonomously when excluded from pollinators. In weedy plants, the proportion of self-compatible individuals was most responsible for producing the highest fruit set in all treatments. It has been reported that self-compatible individuals in cultivated plants of *P. philadelphica* (Pandey, 1957; Labate and Robertson, 2015) result from the S locus mutation (Mulato-Brito et al., 2007) causing an irreversible loss of the SI system (Igic et al., 2008). Instead, *P. philadelphica* has a labile reproductive system (self- and cross-fertilization) and may present a mixed system strategy, allowing fruit production by autogamy and allogamy (Carvalho et al., 2020). In small weedy populations of *P. philadelphica*, self-pollination would provide an advantage assuring some seed reproduction but under crop domestication artificial selection could favor SI individuals in the production of larger flowers and fruits.

### Why Is It More Common to Find SI Individuals in Cultivated Plants of *P. philadelphica* Than in Weedy Plants?

Self-incompatibility in domesticated plants of *P. philadelphica* is atypical compared to the trend in SC in most domesticated species (Dempewolf et al., 2012). *Solanum lycopersicum* and *Capsicum annum*, for example, are SC species under domestication, but their wild populations are SI (Pickersgill, 1997; Bai and Lindhout, 2007; Bedinger et al., 2010). Conversely, it is more common to find SI individuals in cultivars of *P. philadelphica* than in weedy populations. One possible explanation for this is the loss of genetic diversity in domesticated plants compared with their wild relatives due to a bottleneck during domestication (Doebley, 1989; Cox, 2009). For example, the domesticated *S. lycopersicum* genetically depauperate while wild populations are rich genetic reservoirs (Bai and Lindhout, 2007). However, it has been found that genetic diversity of *P. philadelphica* is slightly higher in cultivated plants than in weedy and wild plants (Zamora-Tavares et al., 2015). This apparently contradictory evidence might be due to the higher proportion of self-incompatible individuals found in crops, which favors outcrossing and increases genetic diversity, compared with that of landraces and weedy plants. An example of this can be found in *Arabidopsis lyrata* as research has shown that the SI predominant populations present higher outcrossing rates than those observed in the predominantly SC populations. This shift in the mating system is also associated with a loss of genetic diversity (Mable et al., 2005). Future studies should characterize the mating system of *P. philadelphica* using molecular markers and their relationship to the degree of domestication.

Another possible explanation for this unexpected shift toward SI in cultivated plants of *P. philadelphica* is that SI individuals were artificially selected during the domestication process. Seeds and fruits with more desirable traits were collected by people (Doebley et al., 2006). In the case of SI plants, fruits are usually larger compared with those from SC plants (see above). It is probable that large fruits were artificially selected from SI individuals, inheriting incompatibility via the nucleus (Mulato-Brito et al., 2007). Self-compatible individuals in crops are most likely to appear later due to random mutations of the S locus retained by artificial selection.

### Ecological Advantages/Disadvantages of Changes in *P. philadelphica* Reproductive Strategies

Variation in SI strength could allow SC individuals to reproduce when compatible mates or pollinators are limited (Mable et al., 2005). However, SC has been associated with costs such as inbreeding depression, pollen, ovule and seed discounting, and low genetic variation (Lloyd, 1992; Barrett, 2002). As spontaneous self-pollination ensures reproduction (Eckert et al., 2006), we suggest that SC individuals of *P. philadelphica*, which accept their own pollen by autonomous autogamy, can ensure some fruit production in all degrees of domestication if pollen is limited. This is to be anticipated in small wild populations that are usually ruderal with few individuals (Zamora-Tavares et al., 2015) and when compatible mates are probably limited. In other SI taxa in the Solanaceae, it is common to find SC individuals in small populations (Stone et al., 2006) where S-allele diversity is probably low, and the cost of SI is high (Vallejo-Marín and Uyenoyama, 2004).

Furthermore, we found a high number of self-deposited pollen grains on stigmas (Range = 54–191 pollen grains) in all degrees of domestication, confirming autonomous pollen deposition. This might be associated with the cost of pollen discounting (i.e., the reduction of the outcross pollen pool due to a higher contribution by self-pollinating to seed set) or to seed discounting (i.e., relative changes in the number of seeds set by outcrossing and self-pollination; Lloyd, 1992; Vallejo-Marín and Uyenoyama, 2004). If most of the pollen deposited on the stigma is self-pollen compared to outcross pollen, the seed set will be severely reduced in SI individuals (Vallejo-Marín and Uyenoyama, 2004). Therefore, it is relevant to study pollen deposition and the pollination ecology of *P. philadelphica* undergoing different degrees of domestication.

Germination percentage in self-pollinated wild species of *Physalis* (e.g., *Physalis acutifolia*, *Physalis chenopodifolia*, and *Physalis pubescens*) is high. They also present a high percentage of plant establishment under greenhouse conditions (Valdivia-Mares et al., 2016). In *P. philadelphica*, fruit and seed production by spontaneous self-pollination is similar or higher compared to those in outcrossing treatments in all degrees of domestication, yet fewer seeds are produced. Furthermore, these surviving seeds germinate at a lower or similar percentage than outcrossed seeds, depending on the degree of domestication. Another study reported that *Physalis* seeds show a secondary or induced latency following cold storage, which is more marked in the wild (Sánchez-Martínez et al., 2018). Researchers suggest that this type of latency in wild species provides plants with a mechanism to survive temperature fluctuations, whereas cultivated species have lost this characteristic through domestication. Moreover, Ozaslan et al. (2017) have indicated that *P. philadelphica* seeds germinate under a wide range of environmental conditions including extremes of ambient temperature, pH, water levels and soil salinity. This helps explain the invasive potential of this plant in Turkey.

In weedy populations of *P. philadelphica*, cotyledons and embryonic foliage leaves resulting from spontaneous self-pollination emerged before those of landraces and commercial plants in the same treatment in this study. In addition, in early stages of seedling development (cotyledon emergence) they did present differences in height compared with outcrosses, but then they were surpassed by outcross seedlings a week following germination. The cotyledon and embryonic foliage leaf size in seedlings resulting from autonomous autogamy did not differ from outcrosses, nor did they differ in terms of survival probability and proportion. This suggests that in weedy plants low inbreeding depression in seeds, resulting from autonomous autogamy, is to be expected in partially self-fertilized species (Charlesworth and Charlesworth, 1987). Similar results have been obtained from self-compatible *Physalis angulata*, whose low inbreeding depression index (δ = 0.27) makes self-pollination favorable (Carvalho et al., 2020). Although early emergence of cotyledons and leaves could provide an advantage for plant establishment in weedy plants, seedling germination and growth, reported in this study, occurred in a greenhouse under controlled conditions. There are certain limitations in assessing plant competition under greenhouse conditions. Further research may consider these variables in field conditions taking into account plant density (low in weedy populations vs. high in crops) as well as water and nutrient availability (Gurevitch et al., 2006).

Within the Solanaceae some species in some genera such as *Capsicum* and *Solanum* present unilateral incompatibility (i.e., pollen of an SC population is rejected on flowers styles of an SI population but not in reciprocal crosses). This makes hybridization difficult (Pickersgill, 1997; Bedinger et al., 2010; Samuels, 2012). Unilateral incompatibility represents a barrier between SI and SC populations of the same or different species, and it evolved as a stage in which SI is lost (Bedinger et al., 2010). It seems that in *P. philadelphica* unilateral incompatibility was not present, or it was weak. When weedy, most SC plants donated pollen to commercial SI plants, and higher fruit production occurred (see above). Thus, artificial selection favors the production of larger but fewer fruits compared to their partially SI progenitors (Doebley et al., 2006). The finding in this study shows that crosses between commercial and weedy plants could increase crop productivity probably with desirable phenotypes, as it is the case of domesticated *Helianthus*. When domesticated *Helianthus* was crossed with wild relative, *Helianthus mollis*, their progeny tended to resemble the domesticated parent while progeny of the reciprocal cross favored *H. mollis* phenotype (Faure et al., 2002).

### Possible Implications of Gene Flow Among Different Degrees of Domestication

Cultivated and wild husk tomato distributed mainly throughout Mexico (Montes-Hernández and Aguirre-Rivera, 1994; Zamora-Tavares et al., 2015; SIAP, 2018), and they are sympatric. This might increase the probability of crosses between weedy and domesticated plants, but gene flow has been found to be low (Zamora-Tavares et al., 2015). Gene flow between wild and semi-domesticated plants may ultimately be useful to traditional and commercial crops as it could increase a crop’s genetic diversity in the future. It could also expand its adaptive traits relevant to pest and/or disease resistance and environmental stress (Rick, 1988; Cox, 2009; Warschefsky et al., 2014; Purugganan, 2019). The SI system of *P. philadelphica* is a factor that limits crop improvement. Therefore, it is desirable to find self-compatible genes present in landraces (Mulato-Brito et al., 2007). As reported above, there is a high probability of finding compatible individuals in weedy and landrace populations. However, gene flow in the opposite direction (from crops to landraces and wild populations) might be dangerous in a context involving introduction of transgenic crops, evolution of aggressive weeds, or increased extinction of wild species (Ellstrand et al., 1999; Alavez-Gómez et al., 2009; Velázquez-López et al., 2018). From our findings, it is important to emphasize that fruit and seed set is higher when commercial plants receive pollen from weedy plants and landraces rather than when crossing occurs in the opposite direction. In the field, plants with different degrees of domestication are in sympatry, and thus it is necessary to study pollen flow among them and the survival and identification of their hybrids under field conditions.

### Conservation of *P. philadelphica*

*Physalis philadelphica* could be a suitable model system to explore how artificial selection favors SI in some cultivated plants. It is of relevant importance to conserve the gradient of domestication found in Mexican territories. The conservation should be *in situ* (in ecosystems and crops) and *ex situ* (seed banks and botanical gardens) in order to preserve the genetic reservoir of this species due to its high genetic diversity as reported under different degrees of domestication (Alavez-Gómez et al., 2009; Meyer et al., 2012; Zamora-Tavares et al., 2015).

## Data Availability Statement

The datasets presented in this study can be found in online repositories. The names of the repository/repositories and accession number(s) can be found in the article/Supplementary Material.

## Author Contributions

LS-M: experiment design, data analysis and manuscript writing. LA-C: experiment design and conduction. MV-P: floral morphology characterization. OV-P: seed collection and manuscript reviewing. All authors contributed to the article and approved the submitted version.

## Conflict of Interest

The authors declare that the research was conducted in the absence of any commercial or financial relationships that could be construed as a potential conflict of interest.
