# Supplementary material for: Changes in Reproductive Traits in Physalis philadelphica; An Unexpected Shift Toward Self-Incompatibility in a Domesticated Annual Fruit Crop
Source: Front Plant Sci. 2021 May 21;12:658406. doi: 10.3389/fpls.2021.658406 (PMC8176284; doi:10.3389/fpls.2021.658406)
Supplement: Supplementary file 1 [file Table_1.docx]

**
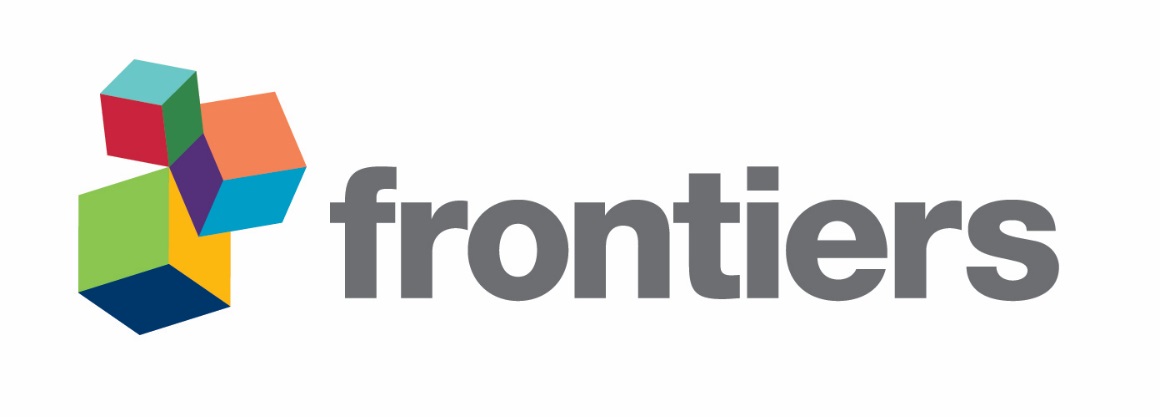
**

Supplementary Table 1 Summary statistics of the seven linear mixed models (LMM). The values in parentheses are the standard errors of the estimate for fixed effects and the standard deviation of the variance for random effects.

| **Variable** | **Estimate (SE)** | **Test statistic (*F*)** | ***P* value** |
| --- | --- | --- | --- |
| Stigmatic area ***Fixed effect*** Degree of domestication ***Random effect*** Individual plant Population | -1.506 (0.345) **Variance (SD)** 0.068 (0.261) 0.444 (0.667) | 0.266 | 0.777 |
| Pollen size ***Fixed effect*** Degree of domestication ***Random effect*** Individual plant Population | 3.075 (0.041) **Variance (SD)** 0.003 (0.056) 0.038 (0.196) | 0.369 | 0.709 |
| Distance stigma-anther ***Fixed effect*** Degree of domestication ***Random effect*** Individual plant Population | 4.856 (0.210) **Variance (SD)** 0.043 (0.207) 0.154 (0.393) | 0.910 | 0.464 |
| Number of pollen deposited on stigma ***Fixed effect*** Degree of domestication ***Random effect*** Individual plant Population | 143.644 (21.050) **Variance (SD)** 477.300 (21.850) 1526.600 (39.070) | 0.945 | 0.450 |
| Number of ovules ***Fixed effect*** Degree of domestication ***Random effect*** Individual plant Population | 5.065 (0.127) **Variance (SD)** 0.048 (0.220) 0.050 (0.223) | 16.028 | 0.007 |
| Number of pollen ***Fixed effect*** Degree of domestication ***Random effect*** Population | 11.804 (0.184) **Variance (SD)** 0.05895 (0.2428) | 2.397 | 0.210 |
| Pollen: ovules ratio ***Fixed effect*** Degree of domestication ***Random effect*** Individual plant Population | 6.918 (0.181) **Variance (SD)** 0.498 (0.223) 0.116 (0.340) | 2.959 | 0.147 |
