# Supplementary material for: Changes in Reproductive Traits in Physalis philadelphica; An Unexpected Shift Toward Self-Incompatibility in a Domesticated Annual Fruit Crop
Source: Front Plant Sci. 2021 May 21;12:658406. doi: 10.3389/fpls.2021.658406 (PMC8176284; doi:10.3389/fpls.2021.658406)
Supplement: Supplementary file 2 [file Table_2.docx]

**
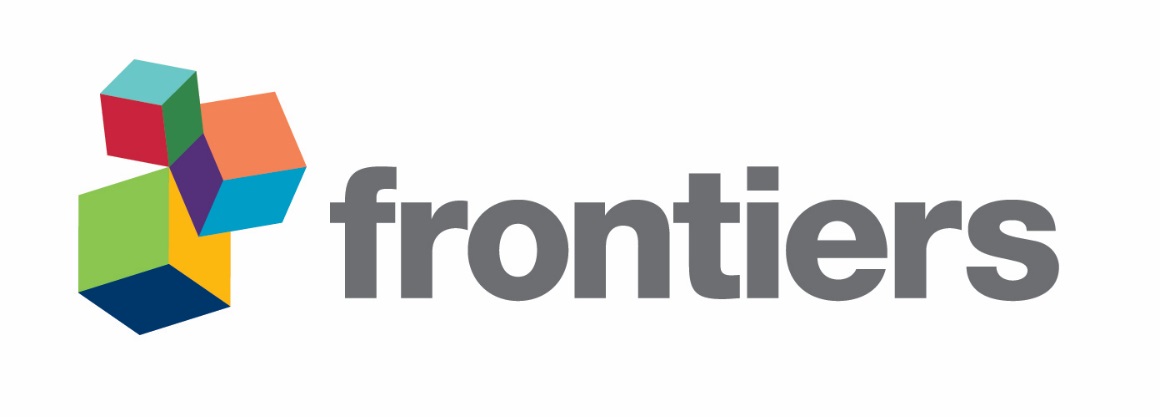
**

Supplementary Table 2 Eigenvectors of the first principal component (PC1) of the principal component analysis of floral morphology traits in *Physalis philadelphica*.

| **Floral trait** | **PC1** |
| --- | --- |
| Length of sepal | 0.8455156 |
| Width of sepal | 0.8602050 |
| Corolla diameter | 0.8522100 |
| Length of anther | 0.6607043 |
| Width of anther | 0.6043218 |
| Length of filament | 0.7211060 |
| Width of filament | 0.6307474 |
| Length of style | 0.8007300 |
| Width of style | 0.5850796 |
| Length of ovary | 0.7710439 |
| Width of ovary | 0.8877125 |
| Length of nectar guides | 0.7476956 |
| Width of nectar guides | 0.7925289 |
